# Effectiveness of a Three-Week Inpatient Pulmonary Rehabilitation Program for Patients after COVID-19: A Prospective Observational Study

**DOI:** 10.3390/ijerph18179001

**Published:** 2021-08-26

**Authors:** Markus C. Hayden, Matthias Limbach, Michael Schuler, Steffen Merkl, Gabriele Schwarzl, Katalin Jakab, Dennis Nowak, Konrad Schultz

**Affiliations:** 1 Bad Reichenhall Clinic, Centre for Rehabilitation, Pulmonology and Orthopedics, 83435 Bad Reichenhall, Germany; markus.hayden@klinik-bad-reichenhall.de (M.C.H.); matthias-peter.limbach@klinik-bad-reichenhall.de (M.L.); steffen.merkl@klinik-bad-reichenhall.de (S.M.); gabriele.schwarzl@klinik-bad-reichenhall.de (G.S.); katalin.jakab@klinik-bad-reichenhall.de (K.J.); 2 Institute for Clinical Epidemiology and Biometry, University of Würzburg, 97080 Würzburg, Germany; michael.schuler@hs-gesundheit.de; 3 University of Applied Science, hs Gesundheit Bochum, 44801 Bochum, Germany; 4 Comprehensive Pneumology Center Munich (CPC), German Center for Lung for Lung Research (DZL), Institute and Clinic for Occupational, Social and Environmental Medicine, Clinic of the University of Munich, 80336 München, Germany; Dennis.Nowak@med.uni-muenchen.de

**Keywords:** COVID-19, pulmonary rehabilitation, course of recovery, inpatient rehabilitation

## Abstract

For COVID-19 patients who remain symptomatic after the acute phase, pulmonary rehabilitation (PR) is recommended. However, only a few studies have investigated the effectiveness of PR, especially considering the duration between the acute phase of COVID-19 and the onset of rehabilitation, as well as the initial severity. This prospective observational study evaluated the efficacy of PR in patients after COVID-19. A total of 120 still-symptomatic patients referred for PR after overcoming acute COVID-19 were asked to participate, of whom 108 (mean age 55.6 ± 10.1 years, 45.4% female) consented. The patients were assigned to three groups according to the time of referral and initial disease severity (severe acute; severe after interval; mild after interval). The primary outcome was dyspnea. Secondary outcomes included other respiratory disease symptoms, physical capacity, lung function, fatigue, quality of life (QoL), depression, and anxiety. Furthermore, patients rated the overall effectiveness of PR and their subjective change in health status. At the end of PR, we detected improvements with large effect sizes in exertional dyspnea, physical capacity, QoL, fatigue, and depression in the overall group. Other parameters changed with small to medium effect sizes. PR was effective after acute COVID-19 in all three groups analyzed.

## 1. Introduction

The clinical course of SARS-CoV-2 infection varies distinctly, ranging from asymptomatic courses [1] to various degrees of severity of coronavirus disease 2019 (COVID-19). In addition to mild and moderate diseases that can mostly be treated on an outpatient basis, severe or critical COVID-19 courses [2] require hospital treatment, admission to an intensive care unit and even ventilation. Particularly after severe or critical courses, i.e., after hospitalization, clinical manifestations often (up to 76% [3,4,5,6]) do not regress after the acute phase of the disease, so many patients still suffer both physically and psychologically from persistent impairments [3,4,5,7]. In addition, although it is less common, even after initially milder courses that could be treated on an outpatient basis, symptoms can persist over time [7,8,9,10,11]. For patients who remain symptomatic after acute COVID-19, rehabilitation is recommended [4,12,13,14]. However, little is known about the efficacy of pulmonary rehabilitation (PR) directly or weeks or months after acute COVID-19 [15,16]. In addition, the optimal timing of PR after COVID-19 has not been clarified. For example, the “COVID-19: Interim Guidance on Rehabilitation in the Hospital and Post-Hospital Phase from a European Respiratory Society and American Thoracic Society coordinated International Task Force” [12] suggests that COVID-19 survivors with a need for rehabilitative interventions at 6 to 8 weeks following hospital discharge should participate in a comprehensive rehabilitation program, whereas in Germany, comprehensive inpatient PR often takes place directly after hospital discharge in the form of a direct transfer from the hospital to a rehabilitation clinic.

To reduce the research gap regarding the efficacy of post-COVID-19 PR, the aims of this study were to assess the pre-post changes in dyspnea, (primary outcome), the most common leading symptom in the context of pulmonary rehabilitation, and other clinically relevant outcomes (secondary outcomes) after PR. In addition, we wanted to analyze which kind of patients were referred to PR by their physicians because of COVID-19. Specifically, we assessed the most burdening symptoms, the time interval after the acute phase, and the initial COVID-19 severity levels.

## 2. Materials and Methods

### 2.1. Setting and Patients

This prospective observational study was conducted at the Bad Reichenhall Clinic (Bad Reichenhall, Germany), an inpatient PR center for adult patients. The study protocol was approved by the ethics committee of the medical faculty of the Ludwig-Maximilians-University Munich, Germany (No. 20-326) and is registered in the German register of clinical studies (DRKS00023180). All patients who were referred to the Bad Reichenhall Clinic because of persistent symptoms after COVID-19 between 28 April 2020 and 8 January 2021 were asked to participate in the study. All referred adult patients (≥18 years) were included, with no age restriction or exclusion criteria except for lack of language or cognitive abilities to fill out questionnaires. Thus, for example, patients requiring oxygen or patients under preexisting nocturnal noninvasive ventilation were also included, as were patients suffering from regularly present comorbidities. The medical services of the insurance providers regularly check the “rehabilitation capability” before granting rehabilitation. Therefore, patients who are still bedridden or receiving invasive ventilation, for example, are not assigned to a PR. All participants provided written informed consent.

### 2.2. Intervention

The three-week inpatient PR program was performed in accordance with the recommendations of the German Respiratory Society for PR in patients with COVID-19 [13]. The program was tailored to each patient’s individual needs and included the following components (O = obligatory for all participants, except those with individual contraindications, F = facultative if needed):

1. *Physical training* (O) consisted of two obligatory main components: (a) *endurance training* was scheduled as 3–5 supervised units per week for 30–60 min each time if this was tolerated by the patient; otherwise, the duration and frequency were adjusted individually. The initial training intensity was based on the individual physical performance orienting on the 6MWD performance for cycle training and was controlled by a modified 0–10 BORG scale [17] (target range 4–6). Accordingly, the training intensities were gradually adjusted by the therapist over the course of the rehabilitation in each session. Exercise intensity was controlled by a pulse oximeter (SpO2 target range ≥90%; oxygen administration if necessary) and heart rate. Endurance-oriented exercises in the terrain (e.g., Nordic walking) and indoor sports were also included. (b) *Strength training* was scheduled for 2–3 supervised sessions per week of 45–60 min each if tolerated; otherwise, the duration and frequency were adjusted individually. Strength training was performed on strength training machines and focused on the major muscle groups (leg press, rowing pull, latissimus pull, butterfly reverse, cable pull, and abdominal exercises). Twelve repetitions in 3 sets were performed until reaching individual local muscular exhaustion at the end of each set. According to this principle, the training intensity was progressively increased. *Whole-body vibration training* (F) was performed 7 times per week and was used only if there was no clinical evidence of thromboembolic complications and if no elevated D-dimer levels were identified. The training consisted of three sets of 1–2-min sessions. Intensities (16–26 Hz, 1.5–4 amplitude) were determined according to the individual perception on the part of the patients. The goal was muscular exhaustion at the end of the set and the unit. The exercise was performed statically in a squat position. *Inspiratory muscle training* (F) was provided for patients with inspiratory muscle weakness (PI max < 7 kPa) and was scheduled for 7 sessions per week for 21 min each, of which 1 session per week was supervised.
2. *Respiratory physiotherapy* (O): 2 units of group respiratory physiotherapy per week for 45 min each session. If necessary, supplemented by (F): (a) *individual breathing training* by physiotherapists, (b) *physiotherapy seminar on coughing techniques*, (c) *mucolytic inhalation therapies* (e.g., NaCl inhalation), and (d) Buteyko breathing exercises.
3. *General physiotherapy* (F), e.g., physiotherapeutic pain management, mobility training or gait training to restore and train everyday functions, respectively, to manage everyday tasks (e.g., climbing stairs) or fascia training.
4. *Patient information* about COVID-19 (O) 45-min presentation by a physician.
5. *Routine medical diagnostics* (O): Medical admission and discharge examination, comprehensive lung function and laboratory diagnostics, blood gas analysis, 6-min walk test [18] with measurement of oxygen saturation, and cardiological function diagnostics (electrocardiogram obligatory, echocardiography if indicated). If necessary, further imaging examinations, psychological, psychiatric, or orthopedic examinations, or other specialist consultations were carried out.
6. *Close medical supervision* (O): All patients received regular medical visits, during which all therapy components, including drug therapy, were reviewed. Oxygen therapy at rest and especially during exertion, e.g., as part of exercise therapy, was available. Noninvasive ventilation could be continued during PR and monitored accordingly.
7. *Psychosocial support*: Psychologically guided self-help group on COVID-19 (O). Social counseling (F) and/or individual psychological counseling (F) and/or neuropsychological diagnostics and training (F) were offered if necessary. Furthermore, relaxation techniques (F), such as progressive muscle relaxation or autogenic training, were offered.
8. *Nutritional counseling* (F) was offered for patients with overweight, diabetes, or other comorbid disorders.
9. *Occupational therapy* (F), e.g., prescription and consultation regarding necessary aids, memory training, or training regarding activities of daily life.

### 2.3. Outcomes and Measures

#### 2.3.1. Primary Outcome

The primary outcome dyspnea was assessed at the beginning (T_1_) and the end (T_2_) of PR based on the current dyspnea sensation and dyspnea in the last 7 days. On the one hand, we used an 11-point numeric rating scale [19] (NRS) to assess the present severity of dyspnea sensation at rest and on exertion. The scales have two dimensions (symptom intensity and symptom unpleasantness) and range from 0 (“no symptoms”) to 10 (“worst imaginable symptom severity”). The minimal clinically important difference (MCID) is considered to be 1 to 2 points [20,21].

On the other hand, the modified Medical Research Council (mMRC) dyspnea scale [22,23,24] was used to rate the impact of dyspnea on daily activities over the last week on a 5-point scale, ranging from 0 (“I only get breathless with strenuous exercise”) to 4 (“I am too breathless to leave the house or I am breathless when dressing”). The mMRC is considered to have low sensitivity to change, and the MCID is considered to be 1 point [25].

#### 2.3.2. Assessment of Secondary Outcomes and Further Variables

Cardinal Symptom

The patients were asked to name their “most important symptom”. To ease the process, we presented a list of symptoms that are frequently associated with COVID-19. Further symptoms could be specified by the patients.

Physical Capacity

The 6-min walking distance (6MWD) was measured using a track length of 30 m according to the European Respiratory Society’s and American Thoracic Society’s technical standards [18]. The deviations from the healthy reference values were calculated according to Enright and Sherrill [26].

Lung Function Tests, Blood Gases, and Laboratory Blood Tests

Forced expiratory volume in one second (FEV1), vital capacity (VC), residual volume (RV), total lung capacity (TLC), total specific airway resistance (sRtot), maximal inspiratory pressure (PImax), and the single-breath transfer factor of the lung for carbon monoxide (TLCO) were determined using spirometry and body plethysmography (MasterScreen Body, CareFusion, Hoechberg, Germany and MasterScreen Diffusion System, Jaeger-Viasys, CareFusion), and respiratory muscle testing was performed using a Jaeger mouth occlusion pressure device in accordance with recommendations of the national guidelines [27,28]. Capillary blood gas samples to assess the partial oxygen pressure (PaO2) and partial carbon dioxide pressure (PaCO2) were taken at rest while breathing ambient air (ABL 800, Radiometer, Willich, Germany). Laboratory chemistry tests, including D-dimers, brain natriuretic peptide (BNP), lactate dehydrogenase (LDH), and C-reactive protein (CRP), were performed by a certified external laboratory (SYNLAB Holding, Augsburg, Germany).

Cough, Sputum, and Pain

Cough, sputum, and pain were assessed with 11-point NRSs, ranging from 0 (“no symptoms”) to 10 (“worst imaginable symptom severity”).

Fatigue

The Brief Fatigue Inventory (BFI [29]) was used to assess fatigue. This questionnaire assesses the severity of and impairment from fatigue with ten questions. The 9 subscales and the total score range from 0 to 10, with higher values indicating higher severity/impairment. Scores of 4 to 6 correspond to “moderate fatigue”, and scores of 7 to 10 correspond to “severe fatigue” [30].

Quality of Life

Generic health-related quality of life (QoL) was measured using the 5-level EuroQol questionnaire (EQ-5D-5L) and the EuroQol visual analogue scale (VAS) [31]. The *descriptive system* of the EQ-5D-5L comprises five dimensions (mobility, self-care, usual activities, pain/discomfort, and anxiety/depression). Each dimension has five levels: “no problems”, “slight problems”, “moderate problems”, “severe problems”, and “extreme problems”. The *VAS* records the patient’s self-rated health on a scale ranging from 0 (“worst possible health condition”) to 100 (“best possible health condition”). The MCID in chronic obstructive pulmonary disease (COPD) patients is 8 points [32].

Depression and Anxiety

The Patient Health Questionnaire-9 (PHQ-9) [33] and the General Anxiety Disorder-7 (GAD-7) [34,35] were used as screening tools to assess symptoms of depression and anxiety. All items are scored on a 4-point Likert scale (0 = “not at all”, 1 = “several days”; 2 = “more than half of the days”; 3 = “nearly all days”). In addition to using the sum of scores (ranging from 0 to 27 for the PHQ-9 and 0 to 21 for the GAD-7), we classified individuals with values of ≥10 as being at risk for a clinically relevant depressive disorder (PHQ-9) or a clinically relevant anxiety disorder (GAD-7).

Estimation of the Overall Effectiveness of Rehabilitation from the Patient’s Perspective

At T_2_, patients were asked to rate the effectiveness of the PR on an 11-point Likert scale, ranging from 0 (“the rehabilitation was ineffective for me”) to 10 (“the rehabilitation was highly effective for me”).

Global Rating of Change in Subjective Health

The global rating of chance in subjective health (GROC) [36] was assessed using one item that compares current subjective health with subjective health at the beginning of rehabilitation and another item that compares current subjective health to subjective health at the time before SARS-CoV-2 infection. The response scale ranges from -7 (“much worse”) to 0 (“no change”) to 7 (“much better”). Changes ≥2 points are considered clinically relevant, and changes ≥4 and ≥6 points are considered moderate and strong, respectively [36,37].

#### 2.3.3. Statistical Analyses

Statistical analyses were performed using *IBM SPSS* (V26) and *R* (V3.6.3). For the analyses of differences between the subsamples at baseline, we used independent-samples *t*-tests for continuous parameters and chi-square tests for categorical variables. For the analyses of changes in the outcomes between T_1_ and T_2_, we used repeated-measures analysis of variance (RM-ANOVA). The evaluations were performed pairwise so that a value was calculated only if both values (T_1_ and T_2_) were available. To assess within-group effect sizes, Cohen’s *d* [38] was calculated by dividing the differences in means between T_2_ and T_1_ by the standard deviation of the change scores. Values of 0.2, 0.5, and 0.8 are considered small, moderate, and large, respectively. We calculated 95% confidence intervals for within-group mean differences as well as for Cohen’s *d*.

Predictors of therapy success were calculated using linear regression analyses. For these analyses, we calculated the changes in clinically relevant variables according to Δ = M_T2_−M_T1_. Intercorrelations between the deltas were calculated using Pearson correlations for parametric variables and Spearman correlations for nonparametric variables.

## 3. Results

### 3.1. Sample

Between 28 April 2020, and 8 January 2021, 120 patients were enrolled in PR due to persistent symptoms after acute COVID-19. One patient was excluded from the study because of cognitive impairment, five patients were excluded because of insufficient German language skills, and six patients did not agree to participate in the study. The remaining 108 patients, consisting of 49 women (mean age: 53.67 ± 10.67 years; mean body mass index (BMI): 30.74 ± 6.64) and 59 men (mean age: 57.20 ± 9.21 years; mean BMI: 29.70 ± 5.54), gave their informed consent and were enrolled. Further baseline descriptive data are displayed in Table 1. One patient dropped out because he withdrew his consent for further study participation. Two patients dropped out because of medical conditions that were not related to PR. The rest of the sample completed the rehabilitation program without complications. The mean treatment duration of PR was 26.3 ± 5.9 days (range: 5–42 days).

The initial acute COVID-19 diagnosis was based on PCR diagnostics in all but one case, for whom the diagnosis was based on antibody detection. All patients were PCR negative upon admission to the clinic.

**Figure 1:**
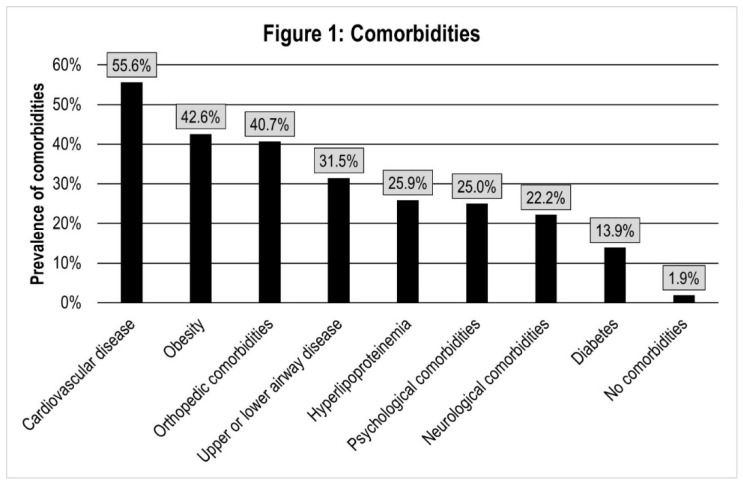
Comorbidities.

Comorbidities were common: Only 2 of the 108 patients (1.9%) had no comorbidities. A total of 55.6% of patients had at least one cardiovascular disease (see Figure 1), most frequently hypertension (52.8%). A total of 31.5% of patients had at least one upper or lower airway disease; among them, 2 patients (1.85%) had COPD, and 18 (16.7%) had asthma. Chi-square tests revealed a statistically significant difference between the groups only for airway diseases, which were more common in group B and C compared to group A (*p* = 0.014). Only 1 patient was a current smoker, 63 (58.3%) had never smoked, and 44 (40.7%) were ex-smokers.

### 3.2. Subsamples

In Germany, PR is usually conducted as a 3-week inpatient intervention in specialized rehabilitation clinics and is a part of the social insurance system. Patients and their treating physicians must apply for rehabilitation with insurance providers. The prerequisite for the approval of PR by German insurance providers is a persistent physical, psychological, and/or social consequence of the illness, for example, persistent physical or psychological sequelae or a threat to the ability to work or to the ability to care for oneself. There are three ways post-COVID-19 patients may be referred to a rehabilitation clinic. First, they may be transferred directly from the hospital, if the medical status requires further inpatient treatment that cannot be granted adequately in ambulant care; second, they can be discharged into home care first and admitted to the rehabilitation clinic after either a few days or after a longer period (a few weeks to several months); and third, they can be admitted to the rehabilitation clinic after having exclusively been treated in an outpatient setting.

Therefore, we clustered the patients according to the mode of admission into three groups (see Table 1). The first group (group A, ‘*acute severe*’) consisted of patients who were admitted to PR either directly after hospital discharge or within one month after discharge from the hospital. This “follow-up rehabilitation” that must be initiated by hospital physicians while the patient is still in the hospital is an integral part of the German rehabilitation system. The second group (group B, ‘*severe after interval*’) consisted of patients who had been hospitalized but were admitted to the PR after more than one month following discharge from the hospital. These rehabilitations were usually not initiated by the hospital but rather through family physicians or specialists in private practice. The last group (group C ‘*mild after interval*’) included patients who had been treated in an outpatient setting or who had been monitored for a maximum of one night in a hospital. These rehabilitations were initiated by physicians in private practice.

Sample characteristics are presented in Table 1. Patients in group C were more likely to be younger *(p* = 0.045) and female (*p* = 0.001) and to less often require oxygen therapy during the acute phase (*p* < 0.001). There were no significant differences between the three subgroups regarding BMI (*p* = 0.265).

### 3.3. Cardinal Symptoms at the Beginning of PR

The most important symptom from the patient’s point of view was dyspnea on exertion. Fifty percent of the whole sample named this symptom the most burdensome. This finding was consistent in all groups. Other symptoms that were stated as the "most important symptom" were anxiety and worries regarding one’s own health status (16.7%), faintness/lack of energy (15.7%), and pain in various parts of the body (7.8%). Chi-square tests of independence did not find any significant difference between the three subgroups (*p* = 0.298 to *p* = 0.918).

### 3.4. Primary Outcome: Dyspnea

**Figure 2:**
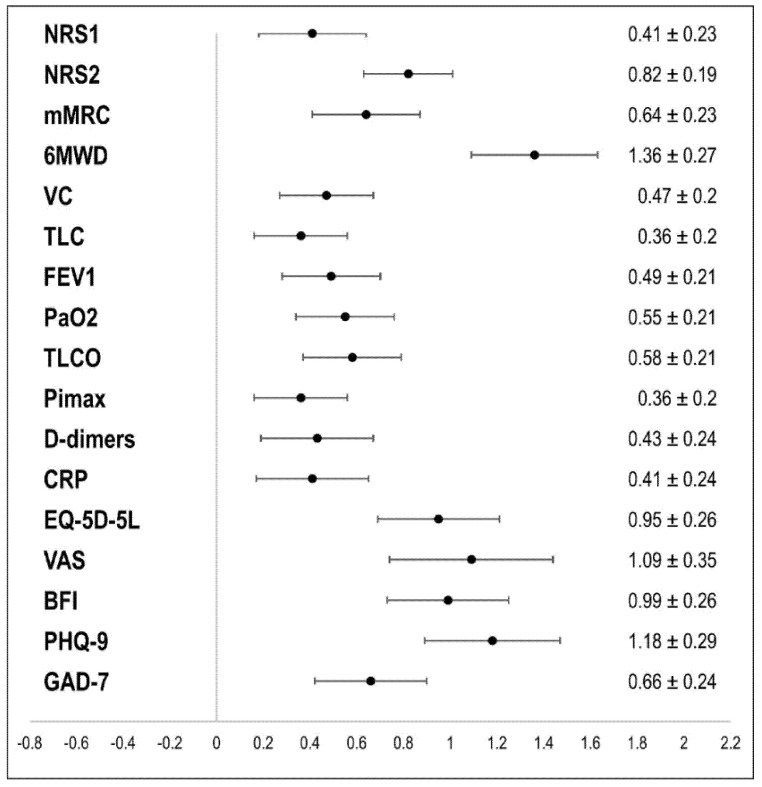
Changes in clinically relevant outcomes over the course of the rehabilitation (absolute value of Cohen’s d and 95% confidence interval). Notes: 6MWD: 6-min walking distance; BFI: Brief Fatigue Inventory; CRP: C-reactive protein; EQ-5D: 5-level EQ-5D questionnaire; FEV1: forced expiratory volume in one second: GAD-7: Generalized Anxiety Disorder-7; mMRC: modified Medical Research Council; NRS1: Numeric Rating Scale for resting dyspnea; NRS2: Numeric Rating Scale for exertional dyspnea; PaO2: partial pressure of O2; PHQ-9: Patient Health Questionnaire 9; PImax: maximal inspiratory pressure; TLC: total lung capacity; TLCO: diffusion capacity of the lungs for carbon monoxide; VAS: visual analogue scale; VC: vital capacity.

The quantitative description of the intensity of dyspnea, as measured by the NRS and the mMRC, is displayed in Table 2, while the NRS results concerning unpleasantness are displayed in Table S1 of the supplement. A graphical illustration of the significant improvements in clinically relevant variables is displayed in Figure 2.

In the overall group, moderate to large pre–post changes were observed for intensity in exertional dyspnea. A clinically relevant improvement of at least 2 points (MCID) was found in 66.1% of patients. There were small to moderate correlations between the improvement in exertional dyspnea and improvements in QoL, fatigue, and anxiety (see Table 3) but not with the improvements in lung function parameters such as VC, TLC, FEV1, PImax, PaO2, or exercise capacity (6MWD).

For the mMRC scores, we also found moderate pre–post changes. Slightly more than 50% benefited from a clinically relevant improvement (MCID = 1 level) without differences between the three subgroups. There were small to moderate correlations between an improvement in mMRC scores and improvements in QoL, fatigue, and anxiety (see Table 3) but not with improvements in lung function or 6MWD.

Small to moderate changes were observed in the intensity of dyspnea at rest, with small correlations between an improvement in resting dyspnea and improvements in 6MWD and anxiety. We found no significant differences between the three subgroups in this regard, either at the start of PR or in the pre-post difference after PR.

### 3.5. Secondary Outcomes: Objective Parameters

#### 3.5.1. Physical Capacity

The 6MWD improved significantly with large effect sizes in all groups, with patients in subgroups A and B showing larger changes than those in group C (see Table 4). There were also significant differences concerning the improvements between the subgroups, indicating that patients who were more severely burdened at T_1_ achieved a higher improvement. Overall, significant reductions in physical performance compared to a healthy reference population matched by age, sex, and BMI [31] were observed. Group A showed the largest deviation of −59.22%pred. Groups B and C showed percentage deviations of −31.41% pred. and −16.45% pred., respectively.

#### 3.5.2. Lung Function

A total of 19.6% of the overall patients had a restrictive lung function pattern (TLC < 80% pred.) at T_1_ (group A 32.7%, group B 8.1%, group C 0%) but only 9.6% at T_2_. In terms of TLC%pred., the total group improved on average by 4.9% (see Table 4). With respect to a reduced VC (VC < 80%pred.), this proportion decreased in the total group from 34.6% at T_1_ to 17.3% at T_2_. The corresponding percentages with VC < 80%pred. at T_1_ in subgroups A, B, and C were 54.6%, 21.9%, and 0%, respectively, and at T_2_ in group A was 12.6% and 12.5% in group B. Lung diffusion, which was slightly impaired at baseline, improved in the overall group, with a moderate effect size. A total of 56.3% of all patients had a pathological lung transfer factor for CO (TLCO single breath <80% pred.) at T_1_ (A = 78.9%, B = 43.8%, C = 15.0%). At T_2,_ the prevalence decreased to 52.1%. At T_1_, group A exhibited significantly greater impairment in several tests of lung function, blood gas and lung diffusion, such as VC, RV, TLC, FEV1, PaO2, and TLCO. Groups A and B showed statistically significant improvements with moderate to high effect sizes in VC%, TLC%, FEV1%, TLCO_SB%, and PImax, while group C, which already showed normal values in this respect at T_1_, showed no improvement in these parameters.

#### 3.5.3. Laboratory Test Results

D-dimers of group A were on average significantly elevated (see Table 4). D-dimers and CRP declined during PR in groups A and B. No changes were observed in BNP or serum LDH levels (see Table S2 of the supplemental material).

### 3.6. Secondary Outcomes: Patient-Reported Outcomes

#### 3.6.1. NRS Cough, Sputum, and Pain

Patients reported only low levels of cough and phlegm at T_1,_ with small to moderate changes in all groups (see Table S1 of the supplemental material). The experience of pain was most pronounced in group C, with small reductions in the overall group.

#### 3.6.2. Quality of Life

Patients in groups A and B showed higher QoL than patients in group C, as measured using the VAS (see Table 5). All groups showed significant improvement with high effect sizes, according to both an increase in the EQ-5D-VAS and a decrease in the descriptive system of the EQ-5D-5L. The MCID of the VAS between the two measurements was 8 points. This was achieved at T_2_ in groups A, B, and C by 81.4%, 70.8%, and 62.5% of patients, respectively. Even though the increase in VAS scores was not intercorrelated with the rating of the general efficacy of the PR (*r* = 0.182, *p* = 0.109), we detected a significant correlation with the first GROC item (improvement compared to the start of rehab) (*r* = 0.360, *p* = 0.001). In the overall group, the improvement in VAS scores correlated significantly with a decrease in mMRC, the intensity of exertional dyspnea, fatigue, and anxiety, and with an increase in the 6MWD.

#### 3.6.3. Fatigue

**Figure 3:**
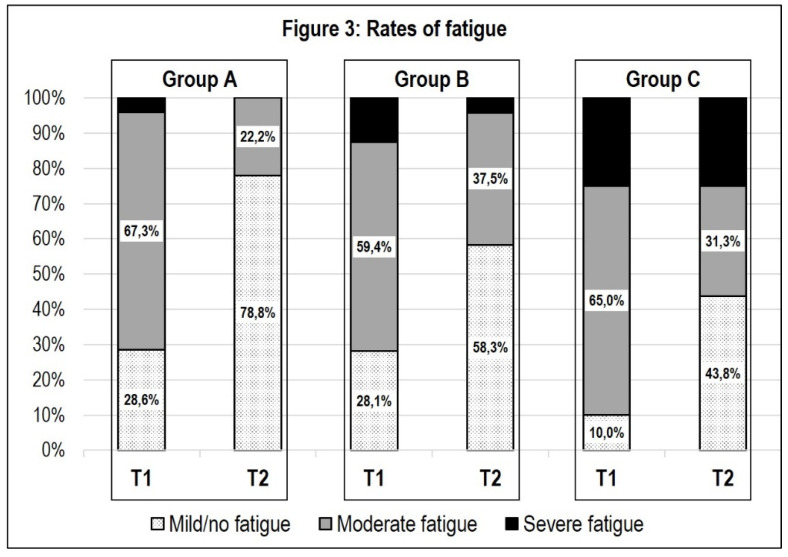
Rates of fatigue.

A significant decrease in fatigue was observed in groups A and B, with large effect sizes. Fatigue was more pronounced in group C at T_1_, where the decrease over the course of PR did not reach statistical significance. To illustrate the findings regarding the differences in fatigue in the three subgroups, the relative frequencies of fatigue severity are displayed in Figure 3.

The overall decrease in fatigue was significantly intercorrelated with decreases in dyspnea, as measured by both the NRS and the mMRC. Furthermore, significant correlations were found for decreases in depression, anxiety, and impairments in QoL.

#### 3.6.4. Depression and Anxiety

At T_1_, the mean values of the PHQ-9 were just below the cutoff between mild and moderate depressive symptom severity (10 points), with 52.6% showing a value of ≥10 and 16.5% showing a value of ≥15, indicating moderately severe depressive psychopathology. Mean values on the PHQ-9 declined with high effect sizes in all groups, and at T_2_, only 22.6% (2.4%) showed values ≥10 points (≥15 points). Anxiety scores at T_1_ indicated, on average, mild psychopathology (5 to 9 points on the GAD-7). However, approximately one-third of the sample (29.3%) showed scores of ≥10. Anxiety values declined, with moderate to high effect sizes. At T_2_ only 8.7% showed values ≥10 points. There were strong correlations between a decrease in fatigue and reductions in both depression and anxiety (see Table 3).

#### 3.6.5. Rating of the Overall Effectiveness of PR from the Patient’s Perspective and Global Rating of Change in Subjective Health

Patients rated the effectiveness of PR as high, ranging from 7.07 in group C to 8.93 in group A (see Table 6). Significant differences could be detected between group A and group C, with group A reporting higher effectiveness of PR.

Regarding GROC values, patients reported, on average, an improvement in health over the course of PR but still reported poorer health when comparing the status at T_2_ with the status before SARS-CoV-2 infection.

*GROC compared to the beginning of PR:* A strong improvement (+6 to +7 points) was reported by 40.0% of group A, 16.0% of group B, and 12.5% of group C, and a moderate improvement (+4 to +5 points) was reported by 46.7%, 52.0% and 37.6% of groups A, B, and C, respectively. A weak but significant correlation was found between the GROC values and the reduction of dyspnea on exertion (*r* = 0.259, *p* = 0.019), the changes in mMRC values (*r* = 0.252, *p* = 0.029), and the increase in the 6MWD (*r* = 0.323, *p* = 0.003). A stronger correlation was found between the GROC values and the reduction in fatigue (*r* = 0.429, *p* < 0.001).

#### 3.6.6. Safety and Feasibility of PR in Patients after COVID-19

During PR, complications were recorded in a systematic and standardized way. Of the 108 patients, one patient had to be transferred to a psychiatric hospital due to an acute psychosocial crisis that was not related to PR. Another patient had to quit PR because of disc prolapse. Another patient terminated study participation prematurely because he withdrew his consent for further study participation. All other 105 patients participated in at least 90% of the physician-prescribed therapies without related side effects.

#### 3.6.7. Prediction of Successful PR Concerning the Primary Outcome of Dyspnea

We did not find significant regression models predicting the reductions in one or both of the primary outcome measures. Further correlation analyses indicated that there were no statistically significant intercorrelations between the reductions in the primary outcome measures (exertional dyspnea NRS and mMRC) and sex, age, BMI, duration of hospitalization, duration of intensive-care treatment, duration of ventilation, or the duration between the acute phase and the beginning of PR. As displayed in Table 3, the decrease in exertional dyspnea was also not correlated with an improvement in the 6MWD or improvements in parameters of the lung function tests or the laboratory measures.

## 4. Discussion

This prospective observational study is the second peer reviewed published study conducted in Germany and, to date, the largest German study to analyze the safety, feasibility, and efficacy of a three-week inpatient PR treatment in patients who were admitted due to persistent symptoms after having gone through acute COVID-19. Ninety percent of the admitted patients agreed to participate. The baseline values of our patient population are broadly comparable to those of another German study [39]. Thus, our patient cohort seems to be representative of post-COVID-19 PR patients in Germany.

### 4.1. Effectiveness of PR

Almost all assessed outcomes improved significantly in each of the analyzed groups at the end of PR, with moderate to large effect sizes. In the whole group, we detected large effect sizes (Cohen’s *d* [38] *d* > 0.8) for the improvement in intensity of exertional dyspnea (primary outcome) and other secondary outcomes, such as exercise capacity, QoL, fatigue, and depression. Regarding effect size, the two strongest improvements were found for the 6MWD (*d* = 1.36) and the VAS scale of the EQ-5D-5L questionnaire (*d* = 1.088), indicating that at the end of PR, patients achieved a significant improvement in physical performance and QoL. Furthermore, moderate effects (0.5 ≤ *d* < 0.8) were found for resting dyspnea and dyspnea in daily life in the last 7 days as measured by the mMRC. Significant but smaller effect sizes (0.2 ≤ *d* < 0.5) were found for cough, sputum, pain, anxiety, lung function parameters (VC, TLC, FEV1, PaO2, TLCO, PI max), and results of the laboratory blood tests (D-dimers, CRP).

Comparing the three subgroups, for some outcomes, stronger effects were found in groups A and B. For example, exertional dyspnea improved in groups A and B with large effect sizes (*d* = 0.922 and *d* = 0.845, respectively), whereas group C only achieved a low to moderate effect size (*d* = 0.485). Similar trends were found for some lung function parameters (VC, FEV1, TLC, PaO2). Furthermore, patients in group C rated both the efficacy of the PR and the personal improvement lower than the other two subgroups. In conclusion, these results indicate that patients with severe forms of acute COVID-19 may show greater improvements over the course of PR, especially if the beginning of treatment occurs soon after the acute phase. These results are in line with the findings obtained by Al Chikhanie et al. [40]. However, it is important to emphasize that significant improvements were also found in group C, with some strong (6MWD, EQ-5D-5L-VAS) and moderate effect sizes (PHQ-9). Cohen’s *d* values >0.4 were also found in group C regarding the intensity of exertional dyspnea, dyspnea in daily life, and impairments in QoL. This indicates that despite a lower effect size, patients with an initially mild course of the disease still benefit from PR even after a long duration, as described by Glöckl et al. [39].

### 4.2. Primary Outcome: Dyspnea

As suspected before the start of the study, exertional dyspnea was stated as the “most important symptom” by most patients in all three groups. The physiological mechanisms of persistent dyspnea in patients after surviving acute COVID-19 have not yet been conclusively elucidated [41,42] but appear to be multicausal. Persistent lung injury and lung function impairments play a significant role [43,44,45]. However, cardiac [46,47], muscular [48], psychological, and other impairments, such as fatigue or deconditioning, may contribute to the development of persistent dyspnea. Some of these impairments can be positively influenced during pulmonary rehabilitation.

Regarding the intensity of exertional dyspnea, as measured by NRS, all three subgroups benefited significantly, with large (groups A and B) or moderate (group C) effect sizes. These improvements were in the same range as those in our recent study on PR for asthma patients. However, the decrease in NRS scores was not associated with improvements in lung function or 6MWD, but there were significant correlations with the reduction in fatigue and especially anxiety (*r* = 0.348). Possibly, the self-confidence in one’s own performance regained through training during PR and the resulting reduction in the fear of exertion contributes to a reduction in exertional dyspnea.

Although most lung function parameters in group C were normal on average and were higher than those in groups A and B, group C scored their resting dyspnea higher at the start of PR and still scored higher at the end of PR. The more pronounced fatigue and the tendentially lower training effects of group C might have had an influence in this regard.

Regarding the mMRC dyspnea scale, there was a significant improvement, with a moderate effect size. This is worth noting because the mMRC dyspnea scale is considered to have low sensitivity to change. The improvement in the mMRC dyspnea scale is descriptively above the mean improvement seen in a recent study on PR for COPD patients [49]. However, similar to the reduction in NRS values, the decrease in mMRC scores was not correlated with an increase in lung function but with a decrease in fatigue and anxiety.

Few post-COVID-19 rehabilitation studies have reported results on dyspnea scores. A dyspnea assessment using the mMRC dyspnea scale was reported for a subgroup in the study by Glöckl et al. [39] (*N* = 26 patients after severe to critical COVID-19). In this study, the mean mMRC at the start of PR was 2. This finding is comparable to the findings in groups A and B, as is the change after PR. Furthermore, the decrease in patients reporting an mMRC score of ≥2 is comparable (77% to 54% in Glöckl et al.; 79% to 54% in our study). In the early rehabilitation group in the study by Curci et al. [50] (*N* = 41 still severely impaired patients, directly transferred from an ICU to the COVID-19 rehabilitation unit of "Policlinico San Marco" Hospital, Zingonia, Bergamo, Italy), which is comparable to our population only to a very limited extent, 90.2% of the patients documented the highest mMRC level at admission. and all improved by at least 1 level. The corresponding figures in group A were 43.9% at rehabilitation onset, of whom 83.3% improved by at least 1 level at discharge. Thus, the changes in mMRC values we detected over the course of PR are comparable to those detected in other studies.

### 4.3. Comorbidities

There was a high burden of comorbidities that exceeds the reports in the literature for the overall group of COVID-19 patients [51,52]; however, it is in line with cross-sectional studies in post-COVID-19 rehabilitation settings [50,53,54,55,56,57]. This difference could be explained by the fact that pre-existing comorbidities negatively influence the course of COVID-19, and patients with a more severe course are more likely to undergo post-COVID-19 rehabilitation. In line with previous research results [52], the most frequently mentioned comorbidities were cardiovascular diseases and obesity. This high prevalence of comorbidities requires the multimodal, multiprofessional, and interdisciplinary approach of PR.

### 4.4. Physical Capacity, Other Clinical Symptoms, and Other Objectively Measured Parameters

A reduced exercise capacity compared to that in the healthy reference population was observed in all patients at T_1_. The largest deviations were found in group A. The observed 6MWD values at T_1_ are in the range of COPD rehabilitants in Germany [49,58] and are slightly above the values for patients suffering from interstitial lung diseases [59]. However, the values may vary with the time interval from the acute phase of the disease and decrease with disease severity, as was shown for post-COVID-19 rehabilitants by Glöckl et al. [39]. This may explain the fact that the 6MWDs of group B and group C were higher than those reported for COVID-19 patients directly after an acute infection [53,54]. In all subgroups, the 6MWD improved with large effect sizes. The changes in groups A and B are descriptively greater than the improvements achieved in our own PR studies concerning COPD or asthma patients [58,60]. The improvement in patients in group C, who started PR a mean of 143 days after the acute phase and whose pulmonary function parameters were normal except for a reduced PImax value, was well above the MCID of patients with COPD or idiopathic pulmonary fibrosis [61,62,63]. There were no significant correlations between the improvement in the 6MWD and lung function parameters except for a correlation with the improvement in PImax. Thus, we assume that the improved exercise capacity might not be a consequence of improved lung function but primarily the result of adaptive mechanisms of the cardiovascular system and the musculature due to exercise training.

### 4.5. Lung Function Tests

The lung function pattern, which was more restrictive at baseline, improved in the overall group with a moderate effect size, with significant improvements in groups A and B. Similar lung function improvements were also seen in some other post-COVID-19 PR studies [39,40,53]. Maximal inspiratory pressure also improved in the total group, with a small effect size, with the most severe limitation and the smallest nonsignificant improvement seen in group C at T_1_. However, the changes in VC, TLC, and FEV1 did not correlate with improvements in exertional dyspnea or physical capacity.

### 4.6. Quality of Life, Fatigue, Depression, and Anxiety

In line with previous research [4,7], we detected not only physical but also mental impairments and subjectively experienced impairments in QoL in the sample at T_1_. In all subgroups, patients reported impairments in QoL and symptoms of fatigue, depression, and anxiety. Over the course of PR, these parameters improved significantly, with mostly large effect sizes. In comparison to the results for depression and anxiety in our own studies on rehabilitants with asthma or COPD [60,64], the results can be considered comparable. Interestingly, both measures of QoL were significantly correlated with a reduction in fatigue. Furthermore, the increase in the EQ-5D-5L-VAS showed a moderate correlation with the reduction in anxiety. However, there were no significant correlations between either of the two measures of the EQ-5D-5L and the decrease in depression. Considering these numbers and the fact that one-third of the patients reported either anxiety and worries regarding their own health status or faintness/lack of energy as their most burdening symptom at T_1_, we assume that fatigue and anxiety are of particular importance in the subjectively experienced burden of disease. Noticeably, improvements in fatigue, anxiety, and QoL were the only variables that showed statistically significant correlations with reductions in exertional dyspnea. Even though these results align with each other, it is currently hard to fully interpret the findings because of a lack of studies that examine the role of anxiety and/or fatigue in PR following COVID-19. Therefore, we highly recommend a closer examination in this regard to further explain our findings.

Regarding the overall subjectively experienced current health status, our data revealed a significant improvement, with large effect sizes for all groups. Three-quarters of the patients achieved an improvement in the VAS score above the MCID of 8 points.

Given the significant reductions in depression and particularly anxiety in all the subgroups, we endorse the conclusion of Demeco et al. [65], who recommended post-COVID-19 rehabilitation programs not only for physical reasons but also for psychological reasons.

Regarding fatigue, we found a significant difference between group C and groups A and B. Group C reported the highest values of fatigue at T_1_ and achieved no significant changes over the course of PR. These results suggest that patients with initially rather mild courses of disease and leading fatigue symptoms may need more specific therapeutic approaches for the treatment of persisting fatigue symptoms. 

### 4.7. Safety and Feasibility of PR in Patients after COVID-19

The rehabilitation program proved to be safe; in particular, no complications occurred, and all but two patients were able to complete PR. Moreover, the program proved to be feasible, since all patients were able to perform more than 90% of the prescribed therapies.

### 4.8. Limitations

There are some limitations that we must point out. First and foremost, the data were from an observational study rather than a randomized controlled trial. Therefore, we cannot state with certainty to what extent rehabilitation caused the improvements. Factors such as the natural course of recovery or regression toward the mean may also have had an influence on the data. Consequently, our results should be interpreted with caution. In order to minimize the research gap, our study group is currently preparing a randomized controlled trial (post-COVID-rehabilitation versus treatment as usual). 

In the current study, patients were classified by duration between the end of acute care and the start of rehabilitation. Future studies could make other group divisions, for example, according to predominant symptomatology or pre-existing conditions. Furthermore, because of the sample size, some effect sizes may not be precisely estimated. This was most prominently observable in the analyses of group C, which only included 21 patients. Therefore, the analyses of this group should be generalized with caution.

It is important to remark that most of the patients assigned to the clinic can be characterized as having an interest in PR and being compliant with treatment recommendations. Furthermore, patients with a positive prognosis for rehabilitation are in general more likely to be admitted to PR. Therefore, the results cannot be easily extrapolated to the total group of all post-COVID-19 patients.

The study relies on data from one clinic only. Although another recently published study of German PR with a comparable patient population found comparable results, possible clinic-specific influences cannot be excluded. Future studies with a multicenter approach would therefore be desirable to validate our findings. Considering the aforementioned limitations, randomized trials are needed to analyze the effects of PR after COVID-19 in more detail.

## 5. Conclusions

In summary, our data suggest that PR might be safe, feasible, and effective in patients after acute COVID-19, thereby improving a variety of clinically relevant outcomes. This seems to be true for all three groups analyzed, with a trend toward greater efficacy after ‘severe courses’ of COVID-19 and an earlier start of rehabilitation after the acute phase of the disease. However, it must be emphasized that significant and clinically relevant effects were also seen after longer intervals of latency following milder courses. Our data suggest that all post-COVID-19 patients who remain symptomatic might benefit from adequate rehabilitation.

## Figures and Tables

**Table 1:** Patient characteristics at baseline.

	Group A “Acute Severe”	Group B “Severe after Interval”	Group C “Mild after Interval”	All Patients
Referral to rehab as	PR after direct transfer from hospital (*n* = 24) or within one month at the latest after discharge (*n* = 31)	PR at the earliest after more than 1 month after discharge from hospital	PR after outpatient treatment (*n* = 16) or monitored in the hospital for a maximum of one night (*n* = 5)	
Number of patients	*N* = 55 (50.9%)	*N* = 32 (29.6%)	*N* = 21 (19.4%)	*N* = 108 (100%)
Age [mean ± SD] (range)	57.9 ± 10.8 (33–85)	54.0 ± 9.9 (32–80)	52.1 ± 6.8 (39–61)	55.6 ± 10.1 (32–85)
Sex [% female]	38.2% (34 M/21 F)	34.4% (21 M/11 F)	81.0% (4 M/17 F)	45.4% (59 M/49 F)
BMI [mean ± SD] (range)	29.9 ± 5.7 (19.3–53.3)	31.5 ± 6.7 (21.0–48.3)	28.9 ± 6.1 (19.9–46.0)	30.17 ± 6.12 (19.3–53.3)
Length of hospitalization [mean ± SD in days] (range)	31.5 ± 18.7 (3–101)	19.4 ± 11.0 (3–49)	Monitored in the hospital for a maximum of one night (*n* = 5)	25.7 ± 17.8 (1–101)
% Oxygen therapy during acute COVID-19 phase	87.3%	68.8%	14.3%	67.6%
% ICU-treatment [mean ± SD in days] (range)	70.9% 21.7 ± 18.1 (4–97)	56.3% 14.5 ± 8.8 (5–40)	-	52.8% 23.6 ± 17.4 (5–97)
% Invasive ventilation [mean ± SD in days] (range)	49.1% 20.0 ± 17.7 (8–87)	37.5% 11.6 ± 4.9 (5–23)	-	36.1% 17.6 ± 15.5 (5–87)
Duration between discharge from the clinic or the acute COVID-19 phase undergone in an outpatient setting and beginning of PR [mean ± SD in days] (range)	10.8 ± 11.2 (0–31)	120.6 ± 70.2 (32–270)	142.9 ± 55.1 (35–270)	69.0 ± 75.3 (0–270)

Notes: BMI: body-mass index; PR: pulmonary rehabilitation; SD: standard deviation.

**Table 2:** Results for the primary outcome measures.

	T_1_		T_2_				
Group	M (Median)	SD (Range)	M (Median)	SD (Range)	Delta [95% CI]	*d* [95% CI]	RM-ANOVA F(df), *p*
**Dyspnea**							
NRS “How intense is your dyspnea at rest?” (☺ 0–10 ☹)							
All patients *N* = 82	1.70 (1.00)	1.82 (0.00–8.00)	1.02 (0.00)	1.49 (0.00–7.00)	−0.67 [−1.03; −0.32]	−0.41 [−0.64; −0.19]	F_T_ = 13.3(1), *p* < 0.001 F_G_ = 2.4(2), *p* = 0.100 F_G*T_ = 0.24(2), *p* = 0.788
A *N* = 43	1.44 (1.00)	1.68 (0.00–8.00)	0.84 (0.00)	1.34 (0.00–7.00)	−0.60 [−1.06; −0.15]	− 0.41 [−0.72; −0.10]	
B *N* = 24	1.63 (1.00)	1.41 (0.00–5.00)	1.00 (1.00)	1.10 (0.00–3.00)	−0.63 [−1.22; −0.03]	−0.49 [−0.86; −0.02]	
C *N* = 15	2.53 (2.00)	2.53 (0.00–7.00)	1.60 (0.50)	2.23 (0.00–7.00)	−0.93 [−2.21; −0.35]	−0.39 [−0.92; 0.13]	
NRS “How intense is your dyspnea on exertion?” (☺ 0–10 ☹)							
All patients *N* = 82	5.56 (6.00)	2.50 (0.00–10.00)	3.41 (3.00)	2.73 (0.00–10.00)	−2.15 [−2.73; −1.57]	−0.82 [−1.01; −0.56]	F_T_ = 39.0(1), *p* < 0.001 F_G_ = 0.25(2), *p* = 0.774 F_G*T_ = 65(2), *p* = 0.524
A *N* = 43	5.60 (5.50)	2.56 (0.00–10.00)	3.23 (2.00)	2.58 (0.00–10.00)	−2.37 [−3.16; −1.58]	−0.92 [−1.28; −0.56]	
B *N* = 24	5.46 (6.00)	2.47 (1.00–9.00)	3.29 (2.00)	2.66 (0.00–8.00)	−2.17 [−3.37; −0.96]	−0.85 [−1.21; −0.30]	
C *N* = 15	5.60 (6.00)	2.53 (0.00–9.00)	4.13 (4.00)	3.29 (0.00–10.00)	−1.47 [−2.87; −0.06]	−0.58 [−1.12; −0.02]	
mMRC (☺ 0–4 ☹)							
All patients *N* = 90	2.26 (2.00)	1.19 (0.00 – 4.00)	1.51 (2.00)	1.12 (0.00–4.00)	−0.74 [−0.99; −0.50]	−0.64 [−0.87; −0.42]	F_T_ = 25.1(1), *p* < 0.001 F_G_ = 1.81(2), *p* = 0.168 F_G*T_ = 1.14(2), *p* = 0.323
A *N* = 48	2.52 (2.00)	1.29 (0.00–4.00)	1.60 (2.00)	1.09 (0.00–4.00)	−0.92 [−1.27; −0.57]	−0.76 [−1.08; −0.44]	
B *N* = 26	1.88 (2.00)	0.82 (0.00–4.00)	1.35 (2.00)	1.23 (0.00–4.00)	−0.53 [−1.04; −0.04]	−0.43 [−0.83; −0.03]	
C *N* = 16	2.06 (2.00)	1.24 (0.00–4.00)	1.50 (1.50)	1.10 (0.00–4.00)	−0.56 [−1.00; −0.13]	−0.69 [−1.23; −0.12]	

Notes: T_1_: start of rehabilitation; T_2_: end of rehabilitation; M: mean; SD: standard deviation; Delta: difference M_T2_−M_T1_; 95% CI: 95% confidence interval; *d*: Cohen’s *d*; RM-ANOVA: repeated-measures analysis of variance; F_T_: F-value for factor “Time” (T_1_ versus T_2_); FG: F value for factor “Group” (A versus B versus C); FG*T: F value for interaction of “Time” and “Group”; NRS: Numeric Rating Scale; mMRC: modified Medical Research Council.

**Table 3:** Correlations between the improvements in various outcomes.

	NRS1	NRS2	mMRC	6MWD	VC	TLC	FEV1	PaO2	TLCO	PImax	D-dimers	CRP	EQ-5D-5L	VAS	BFI	PHQ-9	GAD-7
**NRS1**	1	**0.341** **	0.159	−0.223 *	0.010	0.058	0.143	−0.004	0.141	0.153	-0.014	−0.058	0.134	0.077	0.193	0.196	0.261 *
**NRS2**		1	0.220	0.187	0.159	0.187	0.192	−0.120	0.075	0.073	-0.077	−0.164	**0.363** **	0.273 *	0.242 *	0.204	**0.348** **
**mMRC**			1	0.074	0.099	−0.127	0.092	−0.038	−0.012	0.030	-0.058	−0.050	0.164	**0.405** **	**0.313** **	0.158	0.265 *
**6MWD**				1	0.065	0.058	0.027	0.281 **	0.191	−0.280 **	0.195	0.273 *	0.279 *	0.244 *	0.203	0.124	0.045
**VC**					1	**0.480** **	**0.842** **	0.249 *	0.126	**0.338** **	0.295 *	0.146	0.127	0.118	0.179	0.233^*^	−0.076
**TLC**						1	**0.305** **	0.205 *	0.227 *	0.222 *	**0.305** *	0.108	0.199	−0.020	0.090	0.061	0.014
**FEV1**							1	0.286 **	0.222 *	0.248 *	0.229	0.197	0.124	0.145	0.145	0.290 *	0.077
**PaO2**								1	**0.316** **	−0.068	0.165	0.281	0.006	−0.128	−0.061	0.055	0.017
**TLCO**									1	−0.003	**0.306** *	0.200	0.047	0.010	0.068	0.054	0.066
**PImax**										1	0.173	0.097	−0.021	0.157	−0.064	−0.197	−0.106
**D-dimers**											1	**0.600** **	0.162	0.155	0.062	−0.041	−0.171
**CRP**												1	−0.092	0.102	−0.129	−0.158	−0.118
**EQ-5D-5L**													1	0.296 **	**0.419** **	0.059	0.106
**VAS**														1	0.279 *	0.077	**0.318** **
**BFI**															1	**0.0555** **	**0.498** **
**PHQ-9**																1	**0.473** **
**GAD-7**																	1

Notes: Values in gray indicate nonsignificant results; values in bold indicate correlation coefficients of ≥0.3; *: *p* < 0.05; **: *p* < 0.01; 6MWD: 6-min walking distance; BFI: Brief Fatigue Inventory; CRP: C-reactive protein; EQ-5D: 5-level EQ-5D questionnaire; FEV1: Forced expiratory volume in one second: GAD-7: Generalized Anxiety Disorder-7; mMRC: modified Medical Research Council; NRS1: Numeric Rating Scale for intensity of resting dyspnea; NRS2: Numeric Rating Scale for intensity of exertional dyspnea; PaO2: partial pressure of O2; PHQ-9: Patient Health Questionnaire 9; PImax: maximal inspiratory pressure; TLC: total lung capacity; TLCO: diffusion capacity of the lungs for carbon monoxide; VAS: visual analogue scale; VC: vital capacity.

**Table 4:** Results of physical capacity, pulmonary function, and laboratory blood tests.

	T_1_		T_2_				
Group	M (Median)	SD (Range)	M (Median)	SD (Range)	Delta [95% CI]	*d* [95% CI]	RM-ANOVA F(df), *p*
**Physical Capacity**							
6-MWD [m]							
All patients *N* = 97	419 (450)	127 (13–663)	530 (530)	100 (175–740)	111 [94; 127]	1.36 [1.08; 1.63]	F_T_ = 130.3(1), *p* = 0.001 F_G_ = 5.4(2), *p* = 0.006 F_G*T_ = 4.1(2), *p* = 0.019
A *N* = 52	377 (392)	142 (13–603)	508 (522)	111 (175–720)	131 [107; 155]	1.52 [1.12; 1.92]	
B *N* = 27	459 (470)	79 (270–590)	555 (564)	79.5 (406–675)	96 [72; 120]	1.61 [1.02; 2.17]	
C *N* = 18	480 (479)	96.8 (275–663)	554 (544)	85.9 (380–740)	74 [33; 114]	0.91 [0.35; 1.45]	
**Lung Function**							
Vital Capacity (VC) [%pred.]							
All patients *N* = 103	89.6 (90.9)	20.7 (33.7–130.1)	98.0 (100.3)	16.2 (63.4–129.3)	8.3 [4.9; 11.8]	0.47 [0.27; 0.67]	F_T_ = 9.49(1), *p* = 0.003 F_G_ = 12.0(2), *p* < 0.001 F_G*T_ = 4.77(2), *p* = 0.011
A *N* = 54	81.1 (79.7)	21.7 (33.7–130.1)	93.6 (96.6)	16.5 (63.4–129.3)	12.5 [7.3; 17.7]	0.66 [0.36; 0.95]	
B *N* = 32	95.3 (97.5)	14.4 (63.0–115.5)	102.0 (103.6)	14.5 (64.1–122.7)	6.7 [0.5; 12.8]	0.39 [0.03; 0.75]	
C *N* = 17	106.3 (104.2)	12.9 (85.4–129.6)	104.5 (106.0)	14.9 (68.4–125.5)	−1.8 [−6.27; 2.64]	−0.21 [−0.69; 0.27]	
Total lung capacity (TLC) [%pred.]							
All patients *N* = 103	92.8 (95.6)	17.3 (46.8–134.1)	97.7 (99.1)	14.9 (63.7–128.1)	4.9 [2.3; 7.6]	0.36 [0.16; 0.56]	F_T_ = 5.61(1), *p* = 0.020 F_G_ = 14.8(2), *p* < 0.001 F_G*T_ = 2.47(2), *p* = 0.089
A *N* = 54	85.4 (86.3)	17.0 (46.8–121.4)	92.0 (91.1)	13.8 (63.7–119.6)	6.5 [2.7; 10.4]	0.47 [0.18; 0.74]	
B *N* = 32	98.3 (98.1)	13.9 (70.8–125.7)	104.1 (106.0)	14.8 (72.4–128.1)	5.8 [0.6; 10.9]	0.41 [0.04; 0.76]	
C *N* = 17	105.5 (104.1)	12.0 (81.1–134.1)	103.8 (101.6)	11.1 (81.6–126.9)	−1.7 [−6.9; 3.4]	−0.17 [−0.65; 0.31]	
Forced expiratory volume in in one second (FEV1) [%pred.]							
All patients *N* = 103	92.3 (92.0)	20.8 (23.2–135.7)	100.9 (103.3)	16.5 (36.7–138.1)	8.6 [5.2; 12.0]	0.49 [0.29; 0.70]	F_T_ = 12.6(1), *p* < 0.001 F_G_ = 7.9(2), *p* < 0.001 F_G*T_ = 2.88(2), *p* = 0.061
A *N* = 54	85.0 (85.3)	23.1 (23.2–135.7)	97.2 (97.0)	18.2 (36.7–138.1)	12.16 [6.5; 17.8]	0.59 [0.29; 0.87]	
B *N* = 32	97.3 (96.8)	14.0 (69.1–126.2)	103.7 (106.4)	13.4 (72.5–131.3)	6.4 [1.4; 11.5]	0.46 [0.09; 0.82]	
C *N* = 17	105.8 (106.0)	13.4 (86.7–129.7)	107.3 (108.7)	13.4 (76.6–131.2)	1.5 [−1.6; 4.5]	0.25 [−0.23; 0.72]	
Partial pressure of O2 (PaO2) [mm Hg]							
All patients *N* = 100	74.4 (74.5)	9.0 (43.0–98.0)	78.4 (78.0)	7.1 (58.0–95.0)	4.0 [2.6; 5.5]	0.55 [0.34; 0.76]	F_T_ = 1571.9(1), *p* < 0.001 F_G_ = 4.7(2), *p* = 0.02 F_G*T_ = 6.1(2), *p* = 0.003
A *N* = 51	71.7 (72.0)	9.6 (43.0–88.0)	77.3 (78.0)	7.4 (58.0–94.0)	5.6 [3.7; 7.5]	−0.63 [0.50; 1.14]	
B *N* = 31	75.6 (73.0)	7.2 (66.0–92.0)	79.4 (78.0)	6.6 (70.0–95.0)	3.7 [0.9; 6.5]	0.49 [0.12; 0.86]	
C *N* = 18	79.9 (79.5)	7.3 (69.0–98.0)	80.0 (79.5)	6.7 (71.0–91.0)	0.06 [3.4; −3.5]	0.01 [−0.45; 0.47]	
Diffusion capacity of the lungs for carbon monoxide (TLCO SB) [%pred.]							
All patients *N* = 93	73.8 (77.1)	22.0 (11.7–124.0)	80.0 (78.4)	20.3 (35.1–120.2	6.2 [4.0; 8.4]	0.58 [0.36; 0.79]	F_T_ = 13.4(1), *p* < 0.001 F_G_ = 11.1(2), *p* < 0.001 F_G*T_ = 8.0(2), *p* < 0.001
A *N* = 48	63.6 (61.1)	21.1 (11.7–105.2)	73.4 (73.9)	19.7 (38.0–114.8)	9.9 [6.7; 13.1]	0.91 [0.57; 1.24]	
B *N* = 30	81.4 (84.6)	18.3 (32.7–107.6)	85.2 (82.9)	20.6 (35.1–120.2)	3.8 [0.48; 7.1]	−0.43 [0.05; 0.80]	
C *N* = 15	91.6 (90.7)	12.9 (70.2–124.0)	90.6 (92.4)	14.0 (65.9–118.2)	−1.0 [−5.9; 3.9]	−0.11 [−0.62; 0.40]	
Maximal inspiratory pressure (PImax) [%pred.]							
All patients *N* = 98	62.4 (59.7)	25.9 (6.6–133.3)	70.1 (67.4)	24.7 (19.9–133.3)	7.7 [3.4; 12.0]	0.36 [0.15; 0.56]	F_T_ = 8.85(1), *p* = 0.004 F_G_ = 2.6(2), *p* = 0.083 F_G*T_ = 0.98(2), *p* = 0.38
A *N* = 52	64.5 (63.0)	27.6 (6.6–133.3)	70.9 (68.9)	23.4 (19.9–131.0)	6.3 [1.1; 11.5]	0.34 [0.06; 0.61]	
B *N* = 31	63.5 (61.9)	26.5 (20.1–116.3)	75.5 (69.6)	28.6 (21.5–133.3)	12.0 [1.7; 22.3]	0.43 [0.05; 0.79]	
C *N* = 15	52.4 (54.0)	15.3 (27.7–88.2)	56.1 (53.3)	14.7 (25.8–88.2)	3.7 [−3.2; 10.6]	0.30 [−0.23; 0.81]	
**Laboratory blood tests**							
D-dimers [ng/mL] (Normal value < 500 ng/mL)							
All patients *N* = 69	1082.2 (517.0)	1329.5 (173.0–6824.0)	614.0 (414.0)	578.3 (182.0–2931.0)	−468.2 [-734.9; −201.5]	−0.43 [−0.67; −0.17]	F_T_ = 3.48(1), *p* = 0.07 F_G_ = 3.93(2), *p* = 0.024 F_G*T_ = 5.32(2), *p* = 0.007
A *N* = 41	1490.9 (866.0)	1571.4 (197.0–6824.0)	725.8 (539.0)	611.6 (182.0–2931.0)	−765.1 [−1194.9; −335.2]	-0.56 [−0.89; −0.23]	
B *N* = 18	536.9 (383.0)	502.6 (173.0–2308.0)	516.7 (358.0)	601.5 (213.0–2826.0)	−20.3 [−111.5; 71.0]	-0.11 [−0.35; 0.57]	
C *N* = 10	387.9 (385.5)	108.0 (265.0–583.0)	330.8 (283.5)	127.3 (226.0–571.0)	−57.1 [−146.6; 32.4]	−0.46 [−1.10; 0.21]	
C-reactive protein (CRP) [mg/L] (Normal value < 5.0 mg/L)							
All patients *N* = 71	5.86 (3.70)	5.62 (0.50–27.70)	4.10 (2.80)	4.08 (0.60–20.80)	−1.77 [−2.78; 0.75]	−0.41 [−0.65; −0.16]	F_T_ = 4.8(1), *p* = 0.032 F_G_ = 1.4(2), *p* = 0.251 F_G*T_ = 1.8(2), *p* = 0.178
A *N* = 39	7.01 (4.20)	6.60 (0.80–27.70)	4.47 (2.70)	4.69 (0.60–20.80)	−2.54 [−4.19; 0.90]	0.50 [−0.83; −0.17]	
B *N* = 21	4.93 (3.50)	4.15 (0.90–13.70)	3.69 (3.10)	3.08 (0.60–11.00)	−1.25 [−2.78; 0.29]	−0.37 [−0.81; 0.08]	
C *N* = 11	3.55 (3.10)	2.89 (0.50–8.90)	3.56 (3.00)	3.57 (0.60–12.50)	0.02 [−1.15; 1.18]	0.01 [−0.58; 0.60]	

Notes: 6-MWD: 6-min walking distance test; T_1_: start of rehabilitation; T_2_: end of rehabilitation; M: mean; SD: standard deviation; Delta: difference M_T2_−M_T1_; 95% CI: 95% confidence interval; *d*: Cohen’s *d*; RM-ANOVA: repeated measurement analysis of variance; F_T_: F-value for factor “Time” (T_1_ versus T_2_); FG: F value for factor “Group” (A versus B versus C); FG*T: F value for interaction of “Time” and “Group”.

**Table 5:** Results of patient-reported outcomes.

	T_1_		T_2_				
Group	M (Median)	SD (Range)	M (Median)	SD (Range)	Delta [95% CI]	*d* [95% CI]	RM-ANOVA F(df), *p*
**Quality of life**							
EQ-5D-5L (☺ 5–25 ☹)							
All patients *N* = 82	11.65 (12.00)	3.16 (5.00–20.00)	9.23 (8.50)	3.02 (5.00–16.00)	−2.41 [−2.98; −1.85]	−0.95 [−1.21; −0.68]	F_T_ = 52.6(1), *p* < 0.001 F_G_ = 0.803(2), *p* = 0.452 F_G*T_ = 1.81(2), *p* = 0.169
A *N* = 42	11.60 (12.00)	2.93 (5.00–18.00)	8.67 (8.00)	2.82 (5.00–15.00)	−2.93 [−3.71; −2.15]	−1.17 [−1.56; −0.77]	
B *N* = 25	11.48 (12.00)	3.04 (5.00–17.00)	9.52 (9.00)	2.84 (5.00–15.00)	−1.96 [−2.86; −1.06]	−0.90 [−1.36; −0.42]	
C *N* = 15	12.07 (11.50)	4.06 (6.00–20.00)	10.33 (11.00)	3.62 (5.00–16.00)	−1.73 [−3.44; −0.03]	−0.56 [−1.10; −0.01]	
EQ-5D-5L-VAS (☺ 100–0 ☹)							
All patients *N* = 83	50.01 (50.00)	17.03 (0.00–90.00)	68.05 (70.00)	16.07 (25.00–95.00)	18.04 [14.6; 21.4]	1.09 [0.88; 1.44].	F_T_ = 86.4(1), *p* < 0.001 F_G_ = 5.64(2), *p* = 0.005 F_G*T_ = 1.23(2), *p* = 0.296
A *N* = 43	49.53 (50.00)	17.28 (0.00–90.00)	70.12 (70.00)	15.59 (30.00–95.00)	20.58 [15.7; 25.5]	1.30 [0.89; 1.70]	
B *N* = 24	56.71 (57.50)	12.07 (24.00–80.00)	71.50 (72.50)	12.15 (50.00–90.00)	14.79 [8.45; 21.13]	0.99 [0.49; 1.47]	
C *N* = 16	41.25 (35.00)	19.28 (15.00–80.00)	57.31 (60.00)	18.75 (25.00–85.00)	16.06 [7.92; 24.20]	1.05 [0.43; 1.66]	
**Fatigue**							
BFI (☺ 0–10 ☹)							
All patients *N* = 81	4.39 (4.33)	2.05 (0.00–8.56)	2.69 (2.11)	2.08 (0.00–8.33)	−1.70 [−2.08; −1.32]	−0.99 [−1.25; −0.72]	F_T_ = 54.4(1), *p* < 0.001 F_G_ = 5.76(2), *p* = 0.005 F_G*T_ = 3.41(2), *p* = 0.041
A *N* = 82	4.05 (4.13)	1.88 (0.00–8.00)	1.92 (1.40)	1.43 (0.00–5.56)	−2.13 [−2.59; −1.66]	−1.24 [−1.87; −0.10]	
B *N* = 24	4.47 (4.78)	2.19 (0.22–8.56)	3.03 (2.61)	2.16 (0.11–7.56)	−1.44 [−2.16; −0.72]	−0.85 [−1.31; −0.37]	
C *N* = 15	5.21 (4.56)	2.15 (2.11–8.44)	4.30 (3.78)	2.50 (0.67–8.33)	−0.91 [−2.07; 0.25]	−0.43 [−0.96; 0.10]	
**Depression and anxiety**							
PHQ-9 (☺ 0–27 ☹)							
All patients *N* = 78	9.95 (10.00)	5.28 (0.00–22.00)	5.68 (4.00)	4.20 (0.00–18.00)	−4.27 [−5.09; −3.45]	−1.18 [−1.47; −0.89]	F_T_ = 75.2(1), *p* < 0.001 F_G_ = 1.26(2), *p* = 0.289 F_G*T_ = 1.22(2), *p* = 0.299
A *N* = 42	9.67 (9.00)	5.44 (0.00–22.00)	4.83 (4.00)	3.58 (0.00–14.00)	−4.83 [−5.96; −3.71]	−1.34 [−1.75; −0.92]	
B *N* = 23	9.83 (10.00)	5.11 (1.00–22.00)	6.00 (5.00)	4.34 (0.00–16.00)	−3.83 [−5.39; −2.26]	−1.06 [−1.56; −0.54]	
C *N* = 13	11.08 (11.00)	5.31 (1.00–20.00)	7.85 (6.00)	5.21 (1.00–18.00)	−3.23 [−5.40; −1.06}	−0.90 [0.24; 1.54]	
GAD-7 (☺ 0–21 ☹)							
All patients *N* = 77	6.39 (5.00)	4.92 (0.00–21.00)	4.00 (3.00)	3.83 (0.00–15.00)	−2.39 [−3.22; −1.56]	−0.66 [−0.90; −0.41]	F_T_ = 22.1(1), *p* < 0.001 F_G_ = 0.939(2), *p* = 0.396 F_G*T_ = 1.62(2), *p* = 0.205
A *N* = 40	6.30 (5.00)	5.44 (0.00–21.00)	3.40 (3.00)	3.17 (0.00–12.00)	−2.90 [−4.05; −1.75]	−0.80 [−1.16; −0.44]	
B *N* = 22	6.18 (6.00)	3.80 (0.00–13.00)	3.73 (3.00)	3.97 (0.00–15.00)	−2.45 [−1.07: −0.66]	−0.79 [−1.26; −0.30]	
C *N* = 15	6.93 (7.00)	5.19 (0.00–16.00)	6.00 (7.00)	4.75 (0.00–15.00)	−0.93 [−3.30; 1.43]	−0.22 [−0.73; 0.30]	

Notes: BFI: Brief Fatigue Inventory; EQ-5D-5L: 5-level EQ-5D questionnaire; GAD-7: Generalized Anxiety Disorder-7; PHQ-9: Patient Health Questionnaire 9; VAS: visual analogue scale; NRS: Numeric Rating Scale; T_1_: start of rehabilitation; T_2_: end of rehabilitation; M: mean; SD: standard deviation; Delta: difference M_T2_−M_T1_; 95% CI: 95% confidence interval; *d*: Cohen’s *d*; RM-ANOVA: repeated-measures analysis of variance; F_T_: F-value for factor “Time” (T_1_ versus T_2_); FG: F value for factor “Group” (A versus B versus C); FG*T: F value for interaction of “Time” and “Group”;.

**Table 6:** Patients’ rating of the rehabilitation efficacy.

Group	“How Effective Was the Rehabilitation Program for You?” ☺ 0–10 ☹		“Compared to the Time Just Before I Started Rehabilitation, My Health is Now:” Very Much Worse (−7)–Unchanged (0)–Very Much Better (+7)		“Compared to the Time before My Corona Infection, My Health Is Now:” Very Much Worse (−7)–Unchanged (0)–Very Much Better (+7)	
	M (Median)	SD (Range)	M (Median)	SD (Range)	M (Median)	SD (Range)
All patients	8.28 (9.00)	1.96 0.00–10.00	4.34 (5.00)	2.08 −6.00–7.00	−1.56 −3.00	3.59 −7.00–7.00
A	8.93 (9.50)	1.42 4.00–10.00	5.16 (5.00)	1.48 0.00–7.00	−0.53 −1.00	3.83 −7.00–7.00
B	7.78 (8.00)	1.64 4.00–10.00	4.04 (4.00)	1.37 −1.00–7.00	−1.88 −3.00	2.52 −5.00–5.00
C	7.07 (8.00)	2.87 0.00–10.00	2.81 (3.50)	2.21 −6.00–6.00	−4.25 −5.00	3.01 −7.00–4.00

Notes: M: mean; SD: standard deviation.

## Data Availability

The raw data supporting the conclusions of this article will be made available by the authors, without undue reservation.

## Supplementary Materials

The following are available online at https://www.mdpi.com/article/10.3390/ijerph18179001/s1, Table S1: Results of further patient-reported outcomes, Table S2: Additional results of pulmonary function, and laboratory blood tests.

## List of Abbreviations (in Alphabetical Order)

BFI	Brief Fatigue Inventory
BNP	Brain Natriuretic Peptide
COVID-19	Coronavirus Disease 2019
CRP	c-reactive Protein
EQ-5D-5L	5-level EuroQol Questionnaire
FEV1	Forced Expiratory Volume in One Second
GAD-7	Generalized Anxiety Disorder-7 Questionnaire
GROC	Global Rating of Chance
LDH	Lactate Dehydrogenase
MCID	Minimal Clinically Important Difference
mMRC	Modified Medical Research Council
NICE	National Institute for Health and Care Excellence
NRS	Numerical Rating Scale
PaCO2	Partial Carbon Dioxide Pressure
PaO2	Partial Oxygen Pressure
PCR	Polymerase Chain Reaction
PHQ-9	Patient Health Questionnaire 9
PImax	Maximal Inspiratory Pressure
PR	Pulmonary Rehabilitation
QoL	Quality of Life
RV	Residual Volume
SARS-CoV 2	Severe Acute Respiratory Syndrome Coronavirus 2
sRtot	Total Specific Airway Resistance
T1	Beginning of Pulmonary Rehabilitation
T2	End of Pulmonary Rehabilitation
TLC	Total Lung Capacity
TLCO	Transfer Factor of the Lung for Carbon Monoxide
VAS	Visual Analogue Scale
VC	Vital Capacity
