# Supplementary material for: Effectiveness of a Three-Week Inpatient Pulmonary Rehabilitation Program for Patients after COVID-19: A Prospective Observational Study
Source: Int J Environ Res Public Health. 2021 Aug 26;18(17):9001. doi: 10.3390/ijerph18179001 (PMC8430843; doi:10.3390/ijerph18179001)
Supplement: Supplementary file 1 [file ijerph-18-09001-s001.zip › ijerph-1332287-supplementary.pdf]

# Supplemental material

## Table S1: Results of further patient-reported outcomes

| Group | T1 | | T2 | | | | |
|---|---|---|---|---|---|---|---|
| | M<br>(Median) | SD<br>(Range) | M<br>(Median) | SD<br>(Range) | Delta<br>[95% CI] | d<br>[95% CI] | RM-ANOVA<br>F(df), p |
| **Dyspnea** | | | | | | | |
| NRS "How burdening or debilitating is your dyspnea at rest?" (☺ 0 – 10 ☹) | | | | | | | |
| All patients<br>N = 81 | 2.01<br>(1.00) | 2.28<br>(0.00 – 10.00) | 0.95<br>(0.00) | 1.59<br>(0.00 – 9.00) | -1.06<br>[-1.44; -0.68] | -0.62<br>[-0.86; -0.38] | $F_T = 24.4(1)$,<br>$p < 0.001$ |
| A<br>N = 43 | 1.56<br>(1.00) | 2.03<br>(0.00 – 8.00) | 0.54<br>(0.00) | 0.83<br>(0.00 – 3.00) | -1.02<br>[-1.54; -0.51] | -0.61<br>[-0.93; -0.28] | $F_G = 4.54(2)$,<br>$p = 0.013$ |
| B<br>N = 23 | 2.26<br>(2.00) | 1.89<br>(0.00 – 6.00) | 0.91<br>(1.00) | 1.24<br>(0.00 – 5.00) | -1.35<br>[-2.16; -0.54] | -0.72<br>[-1.17; -0.25] | |
| C<br>N = 15 | 2.93<br>(2.00) | 3.17<br>(0.00 – 10.00) | 2.20<br>(2.00) | 2.78<br>(0.00 – 9.00) | -0.73<br>[-1.61; 0.14] | -0.46<br>[-0.99; 0.08] | $F_{G*T} = 0.601(2)$,<br>$p = 0.551$ |
| NRS "How burdening or debilitating is your dyspnea on exertion?" (☺ 0 – 10 ☹) | | | | | | | |
| All patients<br>N = 81 | 6.03<br>(6.00) | 2.72<br>(0.00 – 10.00) | 3.91<br>(4.00) | 2.93<br>(0.00 – 10.00) | -2.11<br>[-2.70; -1.52] | -0.75<br>[-1.04; -0.54] | $F_T = 33.3(1)$,<br>$p < 0.001$ |
| A<br>N = 43 | 5.79<br>(6.00) | 2.77<br>(0.00 – 10.00) | 3.42<br>(3.00) | 2.58<br>(0.00 – 10.00) | -2.37<br>[-3.18; -1.57] | -0.90<br>[-1.25; -0.54] | $F_G = 1.39(2)$,<br>$p = 0.254$ |
| B<br>N = 23 | 6.29<br>(6.00) | 2.44<br>(1.00 – 10.00) | 3.92<br>(3.00) | 3.06<br>(0.00 – 10.00) | -2.38<br>[-3.59; -1.16] | -0.83<br>[-1.29; -0.36] | |
| C<br>N = 15 | 6.29<br>(6.50) | 3.17<br>(0.00 – 10.00) | 5.43<br>(6.00) | 3.37<br>(0.00 – 10.00) | -0.86<br>[-2.07; 0.36] | -0.41<br>[-0.95; 0.15] | $F_{G*T} = 1.92(2)$,<br>$p = 0.152$ |
| **Cough, sputum, and pain** | | | | | | | |
| NRS "How intense is your cough?" (☺ 0 – 10 ☹) | | | | | | | |
| All patients<br>N = 82 | 1.92<br>(1.00) | 2.37<br>(0.00 – 10.00) | 1.05<br>(0.00) | 1.89<br>(0.00 – 10.00) | -0.87<br>[-1.31; -0.43] | -0.43<br>[-0.66; -0.21] | $F_T = 12.2(1)$,<br>$p < 0.001$ |
| A<br>N = 43 | 1.81<br>(1.00) | 2.09<br>(0.00 – 7.00) | 0.70<br>(0.00) | 0.89<br>(0.00 – 3.00) | -1.12<br>[-1.65; -0.58] | -0.64<br>[-0.96; 0.31] | $F_G = 3.4(2)$,<br>$p = 0.040$ |
| B<br>N = 25 | 1.36<br>(0.00) | 1.98<br>(0.00 – 8.00) | 1.08<br>(0.00) | 2.12<br>(0.00 – 7.00) | -0.28<br>[-0.58; 0.21] | -0.19<br>[-0.58; 0.21] | |

| | | | | | | | |
|---|---|---|---|---|---|---|---|
| C<br>N = 14 | 3.21<br>(2.50) | 3.36<br>(0.00 – 10.00) | 2.07<br>(1.00) | 3.15<br>(0.00 – 10.00) | -1.14<br>[-2.97; 0.68] | -0.36<br>[-0.90; 0.19] | $F_{G*T}$ = 1.57(2),<br>p = 0.214 |
| NRS "How big is the amount of phlegm that you expectorate?" (☺ 0 – 10 ☹) | | | | | | | |
| All patients<br>N = 81 | 1.11<br>(0.00) | 1.80<br>(0.00 – 7.00) | 0.58<br>(0.00) | 1.17<br>(0.00 – 5.00) | -0.53<br>[-0.83; -0.23] | -0.40<br>[-0.62; -0.17] | $F_T$ = 12.6(1),<br>p < 0.001 |
| A<br>N = 42 | 0.91<br>(0.00) | 1.53<br>(0.00 – 5.00) | 0.48<br>(0.00) | 0.86<br>(0.00 – 4.00) | -0.43<br>[-0.86; 0.002] | -0.29<br>[-0.62; 0.001] | $F_G$ = 1.4(2),<br>p = 0.253 |
| B<br>N = 24 | 1.08<br>(0.00) | 1.64<br>(0.00 – 6.00) | 0.50<br>(0.00) | 1.18<br>(0.00 – 5.00) | -0.58<br>[-0.11; -0.07] | -0.48<br>[-0.90; -0.05] | |
| C<br>N = 15 | 1.73<br>(0.00) | 2.60<br>(0.00 – 7.00) | 1.00<br>(0.00) | 1.77<br>(0.00 – 5.00) | -0.73<br>[-1.56; 0.38] | -0.49<br>[-1.02; 0.05] | $F_{G*T}$ = 0.3(2),<br>p = 0.738 |
| NRS "How strong is the pain that you experience?" (☺ 0 – 10 ☹) | | | | | | | |
| All patients<br>N = 83 | 3.46<br>(3.00) | 2.91<br>(0.00 – 10.00) | 2.70<br>(2.00) | 2.68<br>(0.00 – 9.00) | -0.76<br>[-1.33; -0.19] | -0.27<br>[-0.51; -0.07] | $F_T$ = 6.27(1),<br>p = 0.014 |
| A<br>N = 44 | 2.55<br>(2.00) | 2.58<br>(0.00 – 10.00) | 1.75<br>(1.00) | 2.11<br>(0.00 – 8.00) | -0.80<br>[-1.62; 0.03] | -0.29<br>[-0.59; 0.01] | $F_G$ = 8.64(2),<br>p < 0.001 |
| B<br>N = 24 | 4.00<br>(4.0) | 2.83<br>(0.00 – 10.00) | 3.54<br>(4.00) | 2.75<br>(0.00 – 9.00) | -0.46<br>[-1.49; 0.58] | -0.19<br>[-0.59; -0.22] | |
| C<br>N = 15 | 5.27<br>(6.00) | 3.06<br>(0.00 – 9.00) | 4.13<br>(4.00) | 3.09<br>(0.00 – 9.00) | -1.13<br>[-2.61; 0.34] | -0.43<br>[-0.95; 0.11] | $F_{G*T}$ = 0.3(2),<br>p = 0.733 |

Notes: NRS: Numeric Rating Scale; $T_1$: start of rehabilitation; $T_2$: end of rehabilitation; M: mean; SD: standard deviation; Delta: difference $M_{T2} – M_{T1}$; 95% CI: 95% confidence interval; d: Cohen's d; RM-ANOVA: repeated-measures analysis of variance; $F_T$: F-value for factor "Time" ($T_1$ versus $T_2$); FG: F value for factor "Group" (A versus B versus C); FG*T: F value for interaction of "Time" and "Group"

## Table S2: Additional results of pulmonary function, and laboratory blood tests

| Group | $T_1$ | | $T_2$ | | | | |
|---|---|---|---|---|---|---|---|
| | M (Median) | SD (Range) | M (Median) | SD (Range) | Delta [95% CI] | d [95% CI] | RM-ANOVA F(df), p |
| **Lung function parameters** | | | | | | | |
| FEV1/VC [%] | | | | | | | |
| All patients N = 103 | 81.5 (82.6) | 10.4 (45.4 –123.9) | 81.3 (82.8) | 8.2 (39.4 – 107.6) | -0.2 [-2.4; 2.1] | -0.01 [-0.2; 1.7] | $F_T$ = 0.2(1), p = 0.689<br>$F_G$ = 0.3(2), p = 0.721<br>$F_{G*T}$ = 0.7(2), p = 0.481 |
| A N = 54 | 82.3 (83.2) | 12.2 (45.4 – 123.9) | 81.3 (83.1) | 8.99 (39.4 – 107.6) | -1.0 [-5.0; 2.9] | -0.07 [-0.33; 0.19] | |
| B N = 32 | 80.6 (82.1) | 8.7 (56.9 – 98.1) | 80.4 (81.1) | 7.8 (62.4 – 93.8) | -0.3 [-2.6; 2.1] | -0.04 [0.39; -0.31] | |
| C N = 17 | 80.4 (81.4) | 6.0 (66.9 – 88.1) | 83.1 (84.2) | 6.0 (71.9 – 94.7) | 2.8 [0.06; 5.5] | 0.53 [0.01; 1.03] | |
| Specific airway resistance (sRtot) [%pred.] | | | | | | | |
| All patients N=103 | 89.70 (76.7) | 60.0 (39.6 – 600.9) | 81.0 (76.7) | 29.2 (44.2 – 249.1) | -8.7 [-6.3; 3.7] | -0.14 [-0.33; 0.06] | $F_T$ = 1.2(1), p = 0.267<br>$F_G$ = 0.6(2), p = 0.528<br>$F_{G*T}$ = 0.6(2), p = 0.526 |
| A N=54 | 89.1 (74.9) | 78.7 (39.7 – 600.9) | 74.8 (70.8) | 30.7 (44.2 –249.1) | -14.2 [-37.0; 8.6] | -0.17 [-0.43; 0.10] | |
| B N=32 | 89.9 (84.0) | 30.7 (46.9 – 171.5) | 91.7 (85.9) | 26.3 (54.7 – 148.3) | 1.77 [-9.4; 13.0] | 0.06 [-0.29; 0.40] | |
| C N=17 | 91.3 (90.5) | 25.6 (39.6 – 139.2) | 80.3 (82.8) | 24.8 (50.5 – 131.1) | -11.0 [-21.2; -0.7] | -0.55 [-1.05; -0.03] | |
| Residual volume (RV) [%pred.] | | | | | | | |
| All patients N = 103 | 103.3 (102.8) | 27.4 (29.5 – 185.2) | 103.0 (102.0) | 25.4 (44.5 – 176.6) | -0.28 [-5.8; 5.2] | -0.01 [-0.20; 0.18] | $F_T$ < 0.001(1), p = 0.9999<br>$F_G$ = 6.3(2), p = 0.002<br>$F_{G*T}$ = 0.4(2), p = 0.675 |
| A N = 54 | 97.2 (97.3) | 27.1 (55.6 – 185.2) | 95.0 (96.4) | 23.4 (44.5 – 143.5) | -2.2 [-10.5; 6.1] | -0.07 [-0.34; 0.19] | |
| B N = 32 | 109.4 (104.7) | 24.1 (73.8 – 172.9) | 112.8 (110.5) | 26.1 (72.8 – 176.6) | 3.4 [-5.6; 12.3] | 0.14 [-0.21; 0.48] | |
| C N = 17 | 111.4 (111.5) | 30.9 (29.5 – 182.6) | 110.2 (105.0) | 22.8 (62.7 – 157.1) | -1.2 [-15.8; 13.3] | 0.04 [-0.52; 0.43] | |
| Partial pressure of CO2 (PaCO2) [mm Hg] | | | | | | | |
| All patients N = 100 | 36.2 (36.0) | 3.1 (26.0 – 43.0) | (36.0) 36.0 | 3.2 (28.0 – 43.0) | -0.1 [-0.7; 0.4] | -0.06 [-0.25; 0.14] | $F_T$ = 0.4(1), p = 0.549 |

| | | | | | | | |
|---|---|---|---|---|---|---|---|
| A<br>N=51 | 36.2<br>(36.0) | 3.0<br>(28.0 – 43.0) | 36.3 (37.0) | 3.3<br>(29.0 – 43.0) | 0.1<br>[-0.7; 1.0] | 0.05<br>[-0.23; 0.32] | $F_G$ = 0.67(2),<br>p = 0.516<br><br>$F_{G*T}$ = 1.2(2),<br>p = 0.313 |
| B<br>N = 31 | 36.1<br>(36.00) | 2.4<br>(31.0 – 40.0) | 35.3 (36.0) | 2.8<br>(30.0 – 40.0) | -0.8<br>[-1.7; 0.1] | -0.32<br>[-0.68; 0.04] | |
| C<br>N = 18 | 36.5<br>(37.5) | 4.2<br>(26.0 – 43.0) | 36.6<br>(36.5) | 3.7<br>(28.0 – 43.0) | 0.11<br>[-1.4; 1.6] | 0.03<br>[-0.43; 0.50] | |
| **<u>Laboratory blood test</u>** | | | | | | | |
| Lactate dehydrogenase (LDH) [U/l] (Normal value 120 - 240 U/l) | | | | | | | |
| All patients<br>N=56 | 195.8<br>(193.5) | 45.6<br>(100.0 – 327.0) | 191.8<br>(189.0) | 36.3<br>(110.0 – 285.0) | -4.1<br>[-11.9; 3.8] | -0.14<br>[-0.40; 0.12] | $F_T$ = 0.7(1),<br>p = 0.406<br><br>$F_G$ = 1.1(2),<br>p = 0.335<br><br>$F_{G*T}$ = 0.02(2),<br>p = 0.972 |
| A<br>N=30 | 202.2<br>(194.0) | 51.0<br>(121.00 – 327.00) | 198.1<br>(198.0) | 36.5<br>(122.00 – 285.00) | -4.1<br>[-16.9; 8.7] | -0.12<br>[-0.48; 0.24] | |
| B<br>N=17 | 185.2<br>(190.0) | 35.7<br>(100.00 – 250.00) | 180.2<br>(178.0) | 35.1<br>(110.0 – 247.0) | -5.0<br>[-17.2; 7.2] | -0.21<br>[-0.69; 0.27] | |
| Brain natriuretic peptide (BNP) [pg/ml] (Normal value <125 pg/ml) | | | | | | | |
| All<br>N=61 | 203.3<br>(100) | 411.2<br>(35.0 – 2940.0) | 174.9<br>(83.0) | 393.8<br>(35.0 – 2760.0) | -28.4<br>[-100.1; -43.2] | -0.10<br>[-0.35; -0.15] | $F_T$ = 0.23(1),<br>p = 0.632<br><br>$F_G$ = 0.21(2),<br>p = 0.81<br><br>$F_{G*T}$ = 2.46(2),<br>p = 0.094 |
| A<br>N=32 | 314.1<br>(119.0) | 546.9<br>(35.00 – 2940.0) | 263.4<br>(114.5) | 530.7<br>(35.0 – 2760.0) | -50.7<br>[-190.2; -88.8] | -0.13<br>[-0.48; 0.22] | |
| B<br>N=18 | 78.7<br>(83.00) | 31.7<br>(35.0 – 120.0) | 73.50<br>(81.0) | 34.8<br>(35.0 – 145.0) | -5.22<br>[-20.4; 10.0] | -0.17<br>[0.63; -0.30] | |
| C<br>N=11 | 84.7<br>(92.0) | 54.0<br>(35.0 – 181.0) | 83.1<br>(82.0) | 48.8<br>(35.0 – 183.0) | -1.6<br>[-22.7; 19.4] | 0.05<br>[-0.64; 0.54] | |
| C<br>N = 9 | 194.8<br>(195.0) | 44.3<br>(137.0 – 280.0) | 192.7<br>(184.0) | 36.3<br>(150.0 – 269.0) | -2.11<br>[-18.5; 14.2] | -0.10<br>[-0.75; 0.55] | |

Notes: $T_1$: start of rehabilitation; $T_2$: end of rehabilitation; M: mean; SD: standard deviation; Delta: difference $M_{T2} – M_{T1}$; 95% CI: 95% confidence interval; d: Cohen's d; RM-ANOVA: repeated-measures analysis of variance; $F_T$: F-value for factor "Time" ($T_1$ versus $T_2$); FG: F value for factor "Group" (A versus B versus C); FG*T: F value for interaction of "Time" and "Group"
